# Supplementary material for: The ESTMJS (European Society of Temporomandibular Joint Surgeons) Consensus and Evidence-Based Recommendations on Management of Condylar Dislocation
Source: J Clin Med. 2021 Oct 29;10(21):5068. doi: 10.3390/jcm10215068 (PMC8584777; doi:10.3390/jcm10215068)
Supplement: Supplementary file 1 [file jcm-10-05068-s001.zip › jcm-1425706-supplementary.pdf]

Table S1. List of all participating ESTMJS members.

Name	Institute
Participating in Preliminary Vote only	
Mercuri L	Chiago, United States
Sanromán JF	Vigo, Spain
Lehman H	Jerusalem, Israel
Undt G	Vienna, Austria
Participating in Final Vote only	
Allon D	Tel Aviv, Israel
Hirjak D	Bratislava, Slovakia
Cascarini L	London, United Kingdom
Aaggard E	Odense, Denmark
Kolk A	Innsbruck, Austria
Wilms C	Bonn, Germany
Participating in both Preliminary and Final Vote	
Neff A	Marburg, Germany
Sidebottom A	Nottingham, United Kingdom
Speculand B	Birmingham, United Kingdom
Saradin C	Hague/Groningen, the Netherlands
Spallaccia F	Terni, Italy
Monje F	Badajoz, Spain
Spijkervet F	Groningen, the Netherlands
Ulmner M	Stockholm, Sweden
McLeod N	London, United Kingdom
Güven O	Ankara, Turkey
Sembronio S	Udine, Italy
Machoň V	Prague, Czeck Republic
Bonte B	Oostende, Belgium
Ângelo D	Lisbon, Portugal
Saeed N	London, United Kingdom
Gerbino G	Torino, Italy
Participating in Final Vote as Associate Member	
Mommaerts M	Brussels, Belgium
Politis C	Leuven, Belgium

Table S2. List of abbreviations.

**GoR**	**Grade of Recommendation**
SoC	Strength of Consensus
LoE	Level of Evidence

Table S3. Recommendations and votings in preliminary and final vote. GoR1, SoC1: GoR and SoC in preliminary voting. GoR2, SoC2: GoR and SoC in final voting.

Topic	Key Message	GoR1	SoC1	GoR2	SoC2	Changes in Text and Comments
	Examinations					
	sufficient diagnosis based on medical history and physical examination	0	approved by majority	0	full consensus	
	imaging examinations in patients with symptoms allowing for further diagnoses	B	consensus	B	full consensus	X-rays are not mandatory
	optional additional examinations in postacute phase	0	consensus	0	full consensus	
	prosthodontic instrumental functional analysis in case of specific diagnostic questions	0	consensus			DISCARDED
	Conservative Treatment					
	initially, manual reduction as treatment in non-traumatic dislocations	B	approved by majority	B	full consensus	
	manual reduction initially according to the Hippocratic method of reduction	B	approved by majority	B	full consensus	the ESTMJS members have no experience with other methods
	manual reduction initially according to pivot wrist method	B	no consensus			DISCARDED
	manual reduction initially according to either Hippocratic or pivot wrist method	B	approved by majority			DISCARDED
	manual reduction one side at a time	A	no consensus	0	full consensus	or bilaterally
	bite blocks or gloves to prevent biting injury	0	approved by majority	B	full consensus	thumbs on the oblique line instead of the molars
	stabilization of patient's head during manual reduction	B	consensus	B	full consensus	removed: on the headrest
	first attempt to reduce an acute dislocation without medication	0	consensus	0	full consensus	
	further attempts of reduction under medication	0	approved by majority	B	full consensus	
	reduction of unilateral dislocation via extraoral technique	0	approved by majority	0	full consensus	in patients with potential infectious diseases etc.
	after reduction of persisting dislocation, immobilization for 1-4 weeks	B	consensus			DISCARDED
	bandages for stabilization in acute dislocations		NEW	0	full consensus	
	securing methods in cases of recurring, long-standing or persisting/habitual dislocations		NEW	B	full consensus	
	Failure of non-surgical methods before any minimally invasive or open surgical intervention	B	consensus	B	full consensus	
	Flowchart for manual reduction	0	no consensus			DISCARDED
	Minimally invasive Treatment					
	observe warnings of the manufacturer concerning off-label-use of Botulinum toxin	A	consensus	A	full consensus	
	potential indication of Botulinum toxin	B	approved by majority	B	full consensus	
	Surgical treatment					
	surgery only after non-surgical methods remain unsuccessful, e.g. in longstanding dislocations	B	consensus	B	full consensus	
	in recurrent dislocations, surgery only after non-surgical/minimally invasive methods remain unsuccessful	0	approved by majority	B	full consensus	
	especially in persisting dislocations individual approach based on entire range of available methods	B	consensus	B	full consensus	
	Supplementary measures					
	soft diet for a few days after surgery	B	approved by majority	B	full consensus	
	immobilization especially after autologous blood injection or surgery of the capsular ligament complex	0	approved by majority	0	full consensus	
	reconstructive and orthognathic surgery in case of secondary damage, such as malocclusion	0	consensus	0	full consensus	
	Predisposing factors					
	check on possible risk for dislocation prior to any intubation narcosis	B	approved by majority			DISCARDED
	informing patients with risk for dislocation prior to any intubation narcosis	B	consensus			DISCARDED

clinical check of functional jaw mobility before and after intubation	B	consensus	DISCARDED		
General Recommendations					
treatment as early as possible	B	approved by majority	B	full consensus	
treatment for individual patient based on medical history and physical examination	B	consensus	B	full consensus	
